# Stratospheric Ozone destruction by the Bronze-Age Minoan eruption (Santorini Volcano, Greece)

**DOI:** 10.1038/srep12243

**Published:** 2015-07-24

**Authors:** Anita Cadoux, Bruno Scaillet, Slimane Bekki, Clive Oppenheimer, Timothy H. Druitt

**Affiliations:** 1 Université d’Orléans, ISTO, UMR 7327, 45071 Orléans, France; 2 CNRS, ISTO, UMR 7327, 45071 Orléans, France; 3 BRGM, ISTO, UMR 7327, BP 36009, 45060 Orléans, France; 4 Sorbonne Universités, UPMC Université Paris 06 ; Université Versailles St-Quentin; CNRS/INSU, LATMOS-IPSL, France; 5 University of Cambridge, Department of Geography, Downing Place, Cambridge CB2 3EN, United Kingdom; 6 Laboratoire Magmas et Volcans, Université Blaise Pascal—CNRS—IRD, OPGC, 5 rue Kessler, 63038 Clermont-Ferrand, France

## Abstract

The role of volcanogenic halogen-bearing (i.e. chlorine and bromine) compounds in stratospheric ozone chemistry and climate forcing is poorly constrained. While the 1991 eruption of Pinatubo resulted in stratospheric ozone loss, it was due to heterogeneous chemistry on volcanic sulfate aerosols involving chlorine of anthropogenic rather than volcanogenic origin, since co-erupted chlorine was scavenged within the plume. Therefore, it is not known what effect volcanism had on ozone in pre-industrial times, nor what will be its role on future atmospheres with reduced anthropogenic halogens present. By combining petrologic constraints on eruption volatile yields with a global atmospheric chemistry-transport model, we show here that the Bronze-Age ‘Minoan’ eruption of Santorini Volcano released far more halogens than sulfur and that, even if only 2% of these halogens reached the stratosphere, it would have resulted in strong global ozone depletion. The model predicts reductions in ozone columns of 20 to >90% at Northern high latitudes and an ozone recovery taking up to a decade. Our findings emphasise the significance of volcanic halogens for stratosphere chemistry and suggest that modelling of past and future volcanic impacts on Earth’s ozone, climate and ecosystems should systematically consider volcanic halogen emissions in addition to sulfur emissions.

Halogens (especially chlorine and bromine) play important roles in the catalytic destruction of atmospheric ozone^1^,^2^,^3^. However, the role of ‘volcanogenic’ halogens in stratospheric ozone chemistry and climate forcing remains poorly constrained. It is commonly believed that most of the halogens in explosive volcanic plumes do not enter the stratosphere, because they are removed by hydrometeors in the troposphere^4^,^5^. In contrast, volcanic sulfur emissions are known to play a key role in stratospheric ozone change and climate forcing on annual to decadal timescales^6^. As stratospheric sulfate aerosols backscatter solar radiation, they act to cool the Earth’s troposphere and surface^7^. In addition, the surface of sulfate aerosols provides sites for heterogeneous chemical reactions that activate halogen species which destroy ozone^8^,^9^.

In the case of recent large volcanic eruptions (1982 El Chichón eruption, 1991 Pinatubo eruption), the halogen compounds involved in such reactions were sourced from anthropogenic emissions^1^,^10^ (e.g., chlorofluorocarbons). The 1991 eruption of Mount Pinatubo (Philippines) furnished particularly significant insights into the mechanisms, feedbacks and timescales of such processes^1^. However, the Pinatubo magma was relatively poor in halogens compared to other volcanic eruptions. In addition, it occurred while the global atmosphere was still loaded with anthropogenic emissions of organic halogens. This calls into question the general applicability of the conclusions derived from Pinatubo observations and ensuing modelling to past or forthcoming eruptions. In particular, it is not known what would happen with a volcanic event with a different halogen yield occurring under pre-industrial atmospheric conditions. In addition to these uncertainties, recent models have re-evaluated the fraction of explosively-emitted halogens crossing the tropopause, suggesting significantly higher values, up to 25%^11^,^12^, than previously thought. Recent observations bear out this conclusion^13^,^14^,^15^ (with up to 75% transported). These findings hint at a significant role of volcanogenic halogens in stratospheric chemistry. To address this question, we have examined the well-known Minoan event, for which well established petrological and volcanological attributes enable evaluation of the atmospheric impact of halogen-rich eruptions. In general, quantifying the volatile budgets of large explosive eruptions is necessary in order to assess fully the resulting atmospheric perturbations and understand climate and ecosystem impacts at different spatial and temporal scales. For ancient and un-monitored eruptions, however, this requires robust constraints on eruption magnitude (i.e., total mass of magma erupted) and volatile abundances, which are gathered from field and petrological data, respectively.

We have estimated the volatile (S, Cl, F, Br, I) yields of four large silicic Plinian eruptions of Santorini Volcano over the last 200 kyr, including the Minoan eruption of the late Bronze-Age (see Supplementary Note for an overview of relevant volcanology and petrology). Phase-equilibrium experiments have shown that the pre-eruptive magma storage conditions for these eruptions were all very similar^16^. The rhyodacitic to dacitic magmas were stored at T = 850–900 °C and P_total_ ~ 200 MPa, under moderately reduced conditions (ΔNNO = −0.9 to −0.1). Volatiles may be present in magmas in two forms: dissolved in the silicate melt phase, and exsolved in a free fluid phase (gas and/or brine). Pre-eruptive concentrations of volatiles dissolved in Santorini silicic melts were determined by analysis of phenocryst-hosted glass inclusions^16^ (Table 1). The melts were poor in fluorine (~500–800 ppm) and particularly in sulfur (≤100 ppm), but were rich in water and chlorine (5–6 wt% and ~2500–3500 ppm, respectively). Estimated contents of Br and I are 10.2–12.9 and 0.11–0.14 ppm, respectively (Table 1 and Methods). Dissolved CO_2_ abundances have been found to be very low or below detection^17^,^18^.

A key issue in assessing eruptive releases of volatiles is to ascertain whether a fluid phase was present in the reservoir, as it can contain up to 90% of the volatiles in a magma^19^. The pre-eruptive melts were close to saturation with respect to pure water^16^, with chlorine contents similar to, or higher than those of experimentally-studied brine-saturated silicic liquids at 200 MPa and 800 to 860 °C (ref. 20). Santorini’s silicic magmas derive from fractionation of andesitic melts with about 4–6 wt% dissolved water^21^,^22^,^23^. Experiments^24^ show that about 30 wt% crystallization of andesite melts is required to produce silicic derivatives similar to those involved in Santorini eruptions. Owing to the incompatible behaviour of H_2_O in crystallizing magmas (H_2_O remains mostly in the melt until it reaches H_2_O solubility), the water content of the residual melt will exceed the 6–7 wt% H_2_O solubility of silicate liquids at 200 MPa (or less if stored at shallower pressure). This “excess” water is expressed as a free fluid phase into which all other volatile species must partition.

Altogether, the petrological constraints strongly suggest that Santorini’s silicic magmas were saturated with a fluid phase in the reservoir prior to eruption^16^. Mass balance considerations indicate that the amount of such a free fluid is in the range 1–3 wt% (of total magma erupted), but this ignores the accumulation of fluid at the top part of the reservoir, due to the buoyancy of bubbles in magmas. Geochemical data indicate that the apical portion of silicic reservoirs reaches 5–6 wt% free fluid^25^. Magmas are thought unable to hold more than 5–6 wt% fluid, since this represents a percolation threshold^26^, beyond which excess fluid is lost from the system. Importantly, we note that 5 wt% is also the average value reconciling the volatile budget determined by the conventional petrological method and that measured either by remote-sensing spectroscopic techniques or ice-core methods for arc volcanoes^19^.

## Degassing budgets of Santorini Plinian eruptions

For each eruption, we determined the volatile fractions released from the melt by subtracting the concentration of volatile species in the matrix glass (post-eruptive melt) from those of glass inclusions (pre-eruptive melt). This gives the minimum fractions of volatiles released by the eruptions (Methods). The fractions of volatiles released from the silicic melts are 3–41 ppm of sulfur, 56–592 ppm of chlorine, 174–282 ppm of fluorine, 0.2–2.2 ppm of bromine and 0.002–0.02 ppm of iodine (Table 1). Our new data indicate that fluorine strongly partitions into apatite (D_F_^apatite/melt^ = 28–31, compared to D_Cl_^apatite/melt^ = 2.6–3.3; Table 2), which agrees closely with experimental results for silicic melts at 200 MPa and 900–924 °C, saturated with aqueous halogen-bearing fluids (D_F_^apatite/melt^ = 11–44 and D_Cl_^apatite/melt^ = 1.0–4.5; ref. 27). This further corroborates the hypothesis that Santorini’s silicic magmas coexisted with a free fluid phase prior to eruption. In such a scenario, the fluid phase would have contained 10–14 wt% of Cl, 0.2–0.8 wt% of S, 239–262 ppm of F, 178–225 ppm of Br and 12–15 ppm of I (Table 1 and Methods). The presence of a fluid phase therefore dramatically increases the released fractions of all species, except for fluorine, which has a low D^fluid/melt^ (0.3; ref. 28). The fluid phase was potentially a major source of halogens, and especially of Cl, during these eruptions.

To calculate the volatile yield of an eruption (i.e., the mass of each volatile species released during the eruption), it is necessary to know the total mass of magma erupted (the eruption magnitude). Since magnitudes of the pre-Minoan eruptions are not well constrained (Supplementary Note), we focus on the Bronze-Age Minoan eruption. We take the erupted magma volume as 39 km^3^ (ref. 29) (i.e., a mass of 9 × 10^13^ kg; Table 1); however, more recent studies suggest this may be an underestimate (~60 km^3^, ref. 30; 78–86 km^3^, ref. 31; Supplementary Note). In the following, we provide minimum (melt degassing only) and maximum (degassing of melt and fluid) bounds on the volatile yield for the eruption (Table 3 and Supplementary Table 1). In a scenario with melt degassing only, the Minoan eruption would have released 0.34 Tg of S, 50.6 Tg of Cl, 23 Tg of F, 0.1 Tg of Br and 0.002 Tg of I (Table 3). In our ‘melt + fluid’ degassing scenario, which we consider the most likely one, we assume that the pre-eruptive fluid phase represents 5 wt% of the magma, as explained above. This raises the volatile yield to 36 Tg of S, 675 Tg of Cl, 24 Tg of F, 1.5 Tg of Br and 0.07 Tg of I (Table 3). Some fraction of these volatiles must have crossed the tropopause (~14 km a.s.l. at Santorini’s latitude), since the maximum height of the Plinian column has been estimated as 36 ± 5 km (refs 32 & 33). Recent modelling^34^ confirms this value and suggests that the height of the column associated with the last co-ignimbrite phase of the eruption was as high as the Plinian.

## Stratospheric chemistry changes after the Minoan eruption

We use a global, two-dimensional (zonally averaged) chemistry-transport model (Methods) to investigate the potential impact of the Minoan eruption on stratospheric chemical composition, notably ozone. The model has previously been used to study the impact of sulfur injections from large volcanic eruptions^35^,^36^,^37^,^38^ and has demonstrated its ability to reproduce the observed evolution of the volcanic aerosol load and the associated ozone depletion following the 1991 Pinatubo eruption^35^. The surface mixing ratios of the major source gases are set to values representative of a pre-industrial atmosphere^39^ (CO_2_ = 250 ppmv; CH_4_ = 400 ppbv; N_2_O = 200 pptv; CH_3_Cl = 450 pptv and CH_3_Br = 5 pptv). We assume that the Minoan eruption occurred at the beginning of August, as indicated by insect fossils at the base of the tephra^34^,^40^, although the model-calculated ozone depletion is only very weakly dependent on the season of volcanic injection, because volcanic halogens have a stratospheric e-folding lifetime of about 2–3 years. We use the minimum and maximum estimates for S, Cl and Br release (Table 3); the errors on these estimates are reported in Supplementary Methods. As sulfur is weakly scavenged in volcanic plumes^4^,^12^, we assume that all the sulfur reach the stratosphere.

The fraction of halogens entering the stratosphere after an eruption is uncertain. It depends on several volcanological and environmental conditions such as the total mass of halogens emitted at the vent, the latitude of the eruption (i.e., the altitude of the tropopause), and the scavenging efficiency of the gases in the ascending eruptive column by hydrometeors (i.e., water or ice particles formed in the atmosphere), this latter depending notably on atmospheric humidity. The first approach to model this phenomenon suggested the removal of almost all the volcanic halogens in the troposphere through scavenging by condensed water^4^, preventing substantial stratospheric chlorine injection, and consistent with observations after the 1991 Pinatubo eruption. However, the Pinatubo eruption was very different from the Santorini Minoan eruption. The Pinatubo magma contained very little chlorine and its eruption coincided with the passage of a typhoon, which contributed to efficient scavenging of erupted chlorine within the volcanic plume^1^. More recent modelling efforts^11^,^12^ suggest that up to 25% or more of emitted HCl (the most soluble chlorine species), can be present in the stratosphere one hour after eruption. During large Plinian eruptions, the fast ascent of the eruption cloud may lead to the formation of halogen-bearing ice crystals and even solid salt particles (NaCl containing Br), preserving the halogens for stratospheric release^11^, as observed for the El Chichón 1982 eruption, México^41^. Measurements following the 2000 eruption of Hekla (Iceland) show that approximately 75% of the emitted volcanic HCl entered the stratosphere and was still present within the plume 35 h after eruption^13^,^14^. Here, we assume that only 2% of volcanic halogens reach the stratosphere, which is clearly a conservative minimum estimate with respect to the aforementioned observations^14^,^41^ and plume modelling studies^11^,^12^. The eruption is simulated in the model by injecting volcanic sulfur and halogens uniformly between the tropopause and about 35 km at the latitude of Santorini (36.4°N).

**Figure 1:**
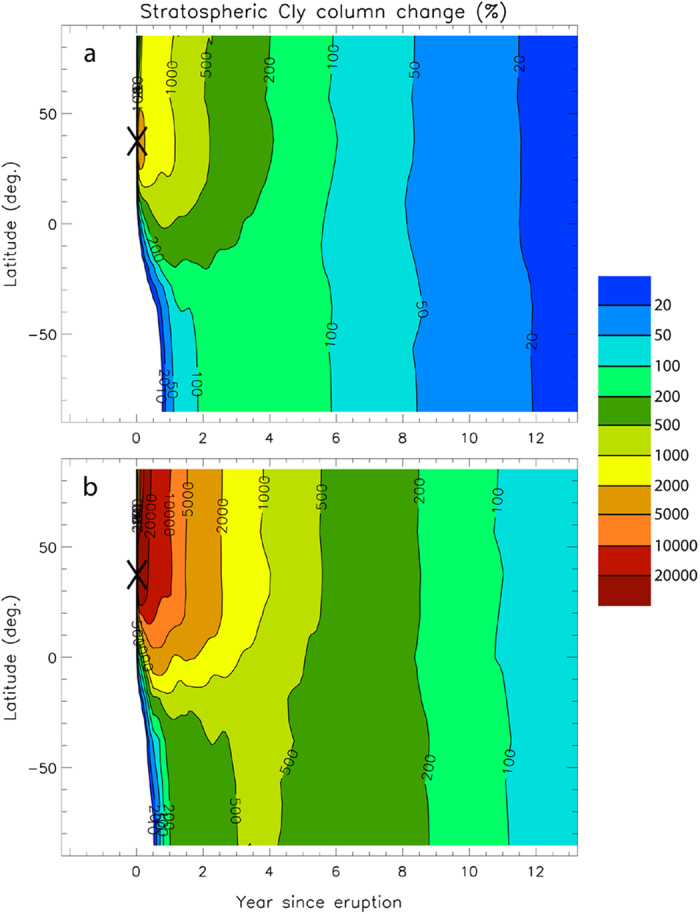
Modelled percentage changes in stratospheric chlorine column following the Minoan eruption as a function of time (years) and latitude. **a** For the minimum volatiles scenario (i.e., no pre-eruptive fluid phase). **b** For the maximum volatiles scenario (i.e., including a pre-eruptive fluid phase). ‘Chlorine column’ = vertically integrated concentration of chlorine. Cly = concentration of total inorganic stratospheric chlorine = HCl + ClONO_2_ + ClO + 2 Cl_2_O_2_ + OClO + 2 Cl_2_ + Cl + HOCl + BrCl. The black cross indicates the latitude of the Santorini eruption.

Figure 1a,b show the modelled percentage changes in the stratospheric chlorine column (vertically integrated concentration of inorganic chlorine) due to the Minoan eruption cloud as a function of time and latitude, using estimates for the minimum and maximum volatile yields, respectively (Table 3). In both simulations, most of the volcanic chlorine is found in the Northern Hemisphere (NH), rather than in the Southern Hemisphere (SH), consistent with typical stratospheric general circulation at mid-latitudes^5^. The modelled chlorine column above Santorini (36.4°N) increases by a factor of 20 to 200 just after the eruption for the minimum and maximum volatile scenarios, respectively. About two years after the eruption, the chlorine column remains 5 (Fig. 1a) to >20 (Fig. 1b) times higher than background (pre-eruptive) columns in the NH, but only 2 to 6 times higher in the SH. About eight years after the eruption, chlorine columns are still 3 times higher than background columns in both the NH and SH in the maximum volatile scenario (Fig. 1b).

**Figure 2:**
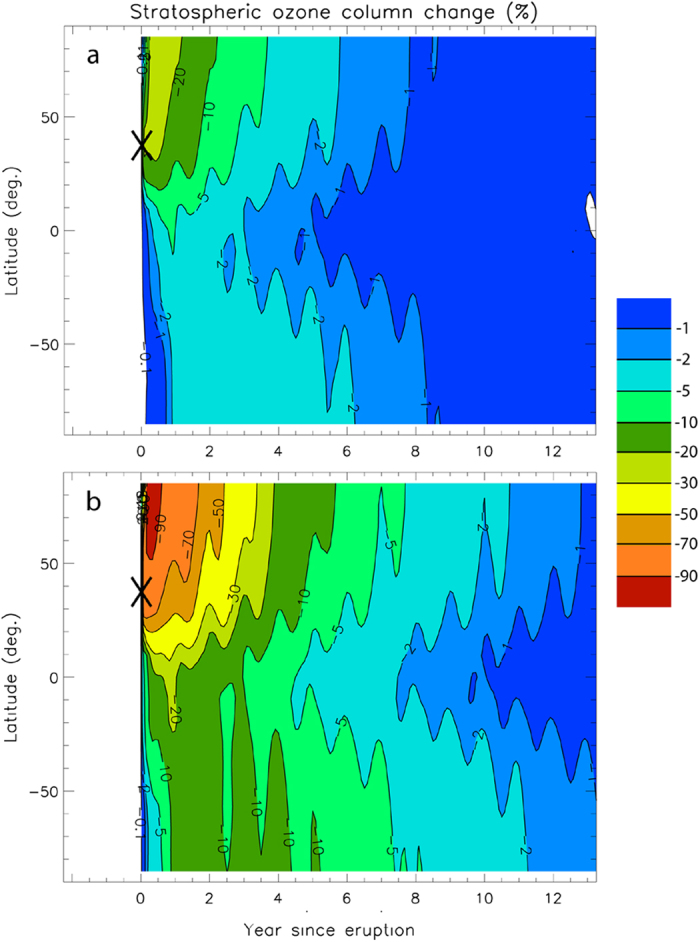
Model-calculated percentage changes in stratospheric ozone column as a function of time (years) and latitude. **a** For the minimum volatiles scenario. **b** For the maximum volatiles scenario. ‘Ozone column’ = vertically integrated concentration of ozone. The black cross indicates the latitude of the Santorini eruption.

Figure 2a,b show the simulated percentage changes in the stratospheric ozone column (vertically integrated concentration of ozone) as functions of time and latitude, again for the minimum and maximum volatile scenarios. Reductions in ozone column are stronger in the NH than in the SH, consistent with the much higher chlorine loading in the NH (Fig. 1a,b). At NH mid-latitudes (Santorini), ozone column losses range from 20% (Fig. 2a) to more than 70% (Fig. 2b) shortly after the eruption. At high NH latitudes, they exceed 20% in the low volatile scenario and 90% in the high volatile scenario. Ozone losses in the SH are about an order of magnitude smaller. Two years after the eruption, ozone columns remain ~30% lower than pre-eruptive columns in the NH and ~10% in the SH in the high volatile scenario (Fig. 2b). Complete stratospheric ozone recovery takes about a decade.

## Volcanic versus anthropogenic halogen emissions

The Minoan eruption discharged prodigious quantities of halogens, in particular Cl and F (51–675 Tg, 23–24 Tg, respectively), but relatively little sulfur (0.34–36 Tg; Table 3). The chlorine output was comparable to that estimated for the mid-10^th^ century eruption of Changbaishan/Mt Paektu, P.R. China/D.P.R. Korea border^42^ (45 Tg of Cl), the 24-kyr-BP Upper Apoyo eruption, Nicaragua^43^ (107 Tg) and the 1815 eruption of Tambora in Indonesia^44^ (211 Tg).

For comparison with organic halogen emissions, the total production of chlorofluorocarbons (CFCs) in 1987 was 1 Tg (ref. 45). The minimum estimate of 51 Tg of chlorine for 39 km^3^ of magma represents a globally averaged increase in chlorine mixing ratio (moles of Cl/moles of air in the atmosphere) of 8 ppbv (parts per billion by volume of air; Table 3). The maximum estimate (675 Tg) amounts to a globally averaged increase in mixing ratio of 106 ppbv (Table 3). For comparison, the anthropogenically driven emissions of chlorine-containing compounds, mostly CFCs, have increased the globally averaged chlorine mixing ratio from 0.55 ppbv (pre-industrial value mostly arising from methyl chloride emissions from the oceans) to 3.8 ppbv in 1995 (i.e., a near seven-fold increase; refs 45 & 46). The stratospheric chlorine abundance has been slowly decreasing since the phase-out of CFC emissions^47^. Hence, even the minimum estimate of chlorine output from the Minoan eruption represents an increment in globally averaged chlorine levels that exceeds the CFCs-generated increment (i.e., ~3 ppbv) by a factor of two.

A corresponding calculation can be made for bromine. The minimum and maximum volatile scenarios represent globally averaged increases in bromine mixing ratios of 8 and 103 pptv (parts per trillion by volume), respectively (Table 3). The rise in anthropogenic emissions of bromine-containing compounds, mostly halons, has increased the stratospheric bromine abundance from about 5 pptv in the pre-industrial period to about 20 pptv by the late 1990s (ref. 5). As for CFCs, it has since been slowly decreasing following the phase-out of halon emissions. The minimum estimate of volcanic bromine emissions represents an increment in globally averaged bromine levels of 3 pptv, i.e., about 20% of the halon-generated increment (i.e., ~15 pptv), while the maximum estimate represents an increment (98 pptv), which exceeds the halon-generated increment by a factor of six.

## Ozone depletion due to volcanic versus anthropogenic halogens

Stratospheric ozone losses predicted by our model in the case of the high volatile scenario (70% to >90% in the NH; Fig. 2b) exceed those observed over Antarctica during austral springs since the 1980s (ref. 48), which are due to the highly-enhanced halogen levels brought by anthropogenic organic Cl- and Br-bearing compounds. This range of ozone depletion also corresponds to future ozone column losses (of about two-thirds) predicted to have occurred by 2065, had CFCs not been phased out under the Montreal Protocol and its amendments (“world avoided” simulation in ref. 49).

The impact of the 1991 Pinatubo eruption on global stratospheric ozone was substantially lower than that predicted for the Minoan eruption, with reductions of the order of 6% in global average total ozone observed by satellites a few months after the eruption^1^. Bekki & Pyle^35^ and Angell^50^ calculated reductions in the total ozone column ranging from 2% in the tropics up to 7% at high-latitudes. Only a very small amount of volcanic chlorine reached the stratosphere, owing to (i) the low abundance of chlorine in the magma (only an estimated 3 Tg of Cl were released^51^), and (ii) the strong wash-out within the plume which was promoted by the wet tropospheric conditions at the time^1^,^51^,^52^.

Our results show that strong ozone depletion can be generated by a volcanic eruption if sufficient halogens reach the stratosphere. This corroborates the hypothesis of Kutterolf *et al.*^43^ that considered the Equivalent Effective Stratospheric Chlorine abundance following large tropical eruptions (EESC is a measure of the ozone destruction potential; EESC = [Cl] _added to stratosphere_ + 60 × [Br] _added to stratosphere_; refs 53, 54, 55). Assuming that 10% of erupted halogen gases reached the stratosphere, Kutterolf *et al.*^43^ calculated EESC values from 23 to 12,770 ppt for Nicaraguan Plinian eruptions, the highest value corresponding to the largest 24 kyr BP Upper Apoyo eruption. The corresponding EESC for the Minoan eruption is 5,600 ppt (minimum scenario) to 74,700 ppt (maximum scenario) (Table 3). Compared with an assumed pre-1980 mean stratospheric background of 900 ppt EESC^55^, the Minoan impact represents an increase in EESC by a factor of 6 to 83. The estimated EESC for the Upper Apoyo eruption represents an increase by a factor of 14 (ref. 43).

Our model also shows that ozone depletion occurs shortly after the Minoan eruption (Fig. 2) and predicts that complete recovery takes about a decade. This contrasts with the 70 years predicted for total replenishment of ozone lost in Antarctica due to CFCs since 1980 (ref. 5). Whereas CFCs have lifetimes of up to a century, inorganic chlorine species (such as HCl) injected into the stratosphere have an e-folding lifetime of 2–3 years. As a result, the return to unperturbed ozone levels following volcanic halogen injections is much faster than the expected ozone return following the phase out of CFC emissions^5^.

## Implications for past and future volcanic impacts on climate

Our models for the Minoan eruption indicate that substantial ozone reductions (20% to >90%; Fig. 2) of the pre-industrial stratosphere (i.e., free of anthropogenic halogen) would have occurred, even if only a few percent of erupted halogens crossed the tropopause (and even with the minimum estimate of erupted magma volume we used to compute volatile budgets). These results demonstrate the important role of volcanogenic halogens on stratosphere chemistry. These stratospheric ozone losses might have generated a significant climate forcing. Ozone is a radiatively important gas as it absorbs both solar ultraviolet and thermal infrared radiation. Stratospheric ozone depletion generated by CFC emissions is estimated to have acted to cool the Earth surface, i.e., it has a negative radiative forcing (RF), reinforcing the radiative effects of volcanic sulfate aerosol. Recent estimates for past stratospheric ozone forcing range between −0.03 and −0.11 W.m^−2^, with a mean of about −0.05 W.m^−2^ (refs 5 and 56, 57, 58, 59). This ozone depletion RF has offset some of the warming from increased greenhouse gas levels over the last few decades. This suggests that, in order to determine accurately the climatic impact of the Minoan (Supplementary Discussion) and other pre-industrial explosive eruptions, future work should consider the ozone depletion RF, in addition to the sulfate aerosol RF.

Our findings highlight the wide variation in volatile yields of volcanic eruptions, and suggest that reconstruction of past impacts of eruptions on ozone, climate and ecosystems (including efforts based on ice-core glaciochemistry; Supplementary Discussion) should consider halogen budgets in addition to that of sulfur, on a case-by-case basis. We stress that our low-halogen scenario for the Minoan eruption (Table 3) could equally correspond to an eruption ten times smaller than the Minoan, but fluid-saturated. Such smaller-magnitude events (e.g., similar to the 1991 eruption of Pinatubo or the 1980 eruption of St Helens) have a return period of a century or less^60^,^61^. If occurring in rapid succession they could affect the environment at the global scale and perturb the recovery of global and Antarctic ozone.

## Methods

The samples studied in this work are the same pumice clasts as those studied by Cadoux *et al.*^16^; they come from the Plinian fallout deposits of each of the selected eruptions, and they are therefore representative of magma near the top of the reservoir.

### Volatile contents in melts and apatites

H_2_O, Cl, F and S contents in plagioclase- and pyroxene-hosted glass inclusions were measured by Secondary Ion Mass Spectrometry (CRPG, Nancy, France), whereas Cl, F and S contents of the matrix glasses were determined by electron microprobe. Analytical procedures and results can be found in ref. 16. The average volatile compositions are reported in Table 1. We estimated bromine contents in both glass inclusions and interstitial glasses by assuming a Cl/Br ratio of 273, which is the average ratio in the continental crust^62^ and which also roughly corresponds to the average ratio in erupted calc-alkaline magmas (Cl/Br = 150–600; ref. 63). We also tentatively estimated the melt iodine contents by assuming an average Br/I ratio of 90 (Br/I = 30–200 in calc-alkaline magmas^63^).

Numerous euhedral apatites occur as inclusions in pyroxenes. CaO, P_2_O_5_, SiO_2_, Cl and F contents (wt%) in apatites were measured by electron microprobe (Cameca SX 100, Clermont-Ferrand, France) with a defocused (10 μm) beam, a sample current of 15 nA and 15 kV acceleration voltage. Counting times were 10 s on peak for Ca, P and Cl and 20 s on peak for Si and F. Sulfur analyses were performed later with a Cameca SX Five microprobe (ISTO, Orléans, France) at 15 nA–15 kV, and with a focused beam; Ca and P were also re-analysed during this session. The standards used were an apatite for Ca and P calibration and a barite (BaSO_4_) for sulfur. Ca and P were measured on PET and TAP crystals, respectively, and S on a large PET crystal. Counting times were 10 s on peak and 5 s on background for Ca and P, and 60 s on peak and 30 s on background for S. The measured S contents (≤0.03 wt%; Table 2) are close to, or lower than, the electron microprobe detection limit (~106 ppm) and thus should be considered as estimates. The very low S contents in Santorini apatites require analytical methods such as SIMS or LA-ICP-MS in order to be accurately determined.

### Degassing calculations

For each eruption, we have first determined the volatile fractions released from the melt and from a fluid phase, respectively. The volatile fractions released from the melt were obtained by subtracting the concentration of volatile species in the matrix glass (post-eruptive degassed melt) from those in glass inclusions (pre-eruptive undegassed melt); this is the ‘conventional’ petrological method. The concentrations of the volatile species in the fluid phase were estimated by different methods, taking into account the pre-eruptive storage conditions of the silicic magmas^16^.

The sulfur content of the fluid phase was estimated using the method of Scaillet and Pichavant (refs 64 & 65), which solves homogeneous equilibria in the C-O-H-S system^66^ of a fluid phase in equilibrium with the silicate melt. Standard thermodynamic considerations show that, at any fixed P and T, if the fugacities of three species are known, then the fugacities of all remaining important species are fixed as well. In our case, the three known fugacities are *f*O_2_, *f*H_2_O and *f*S_2_. The *f*O_2_ is calculated from the oxybarometer of ref. 67, while *f*H_2_O is retrieved from the fugacity-composition (i.e., melt water content) relationships of ref. 68, since at equilibrium *f*H_2_O_gas_ = *f*H_2_O_melt_. The *f*S_2_ is calculated from the model of ref. 65, which uses melt composition (including H_2_O and S contents), at fixed P, T and *f*O_2_. The calculations were performed for the pressure range 150–250 MPa, as inferred from phase equilibrium constraints for the four main Plinian events of Santorini over the last 200 ky (ref. 16). The pressure was adjusted so as to yield melt CO_2_ contents below 300 ppm (calculated using the model of ref. 69), constrained by measurements of silicic glass inclusions in Plinian products at Santorini^17^,^18^. Since both *f*H_2_O and *f*S_2_ depend on total pressure (albeit slightly), the calculations are performed iteratively, until convergence is obtained. Calculations for all four eruptions for which key petrologic information is available yielded pressures in the range 140–260 MPa, and resulted in S content in the fluid phase varying between 0.2 and 1.4 wt%. For the Minoan eruption, the retrieved S content varies between 0.5 and 1.4 wt%. Increasing pressure, everything else being equal, decreases the content in S of the fluid phase by about 30% for every 50 MPa added. For instance, a 50 MPa pressure increase (from 200 to 250 MPa) decreases S content from 0.8 wt% to 0.5 wt%. This would increase the dissolved CO_2_ content from <100 ppm to over 300 ppm. This is the main source of uncertainty on storage conditions (pressure is known to ±40 MPa). Overall, we estimate that our calculated fluid S contents are known to within 30%.

The concentration of chlorine in the fluid was calculated from Webster *et al.*’s partition experiments^27^ at ~200 MPa and 900–924 °C (i.e., at conditions similar to that of the Santorini pre-eruptive magmas^16^) between silicic melts, apatite and Cl-bearing aqueous fluid(s). The resulting data are consistent with a linear increase in the mole fraction of chlorapatite (X^apatite^_Cl_) with increasing Cl in the fluid(s), which gives the following relationship: X^apatite^_Cl_ = 0.011 × wt% Cl in fluids (Table 2).

For fluorine, we used the fluid/melt partition coefficient of 0.3 measured in ref. 28 for peraluminous rhyolitic melts at 200 MPa and temperatures near 800 °C. Bromine and iodine abundances in the fluid were estimated using the fluid/melt partition coefficients (17.5 and 104, respectively) of Bureau *et al.*^62^, who performed partition experiments between silicic (albitic) melt and Br- and I-bearing hydrous fluids at 200 MPa and 900 °C.

To obtain the volatile yields (i.e., the masses of volatiles erupted), the volatile fractions released from melt and fluid phases (Table 1) were then scaled to the mass of melt and the mass of fluid, respectively, using the total erupted mass of magma (calculated from dense magma volume and magma density; Supplementary Table 1). We calculated volatile yield from (i) melt degassing only (minimum estimates) and (ii) from melt and a fluid phase (maximum estimates). Maximum estimates were obtained by adding the contribution of a fluid phase representing 5 wt% of the total erupted mass of magma (this value is explained in the introduction of the main text).

Bromine yields to the atmosphere were estimated in two ways (Supplementary Table 1): (i) Indirectly, by estimating the Br contents of glass inclusions and matrix glass assuming a Cl/Br ratio of 273 (refs 62 & 63), and then applying the procedure described above (i.e., calculation of Br abundance in the melt and in the fluid, then scaling to the mass of erupted magma). (ii) Directly, by multiplying the chlorine yields by the mean molar Br/Cl ratio of 0.0022 in condensate samples from basaltic to rhyolitic magma of 18 arc volcanoes^70^. The Br yields obtained by these two methods are similar (Supplementary Table 1). We choose the results from the second method for our modelling because they are, in our opinion, the most reliable. Besides, this arc average Br/Cl of 0.0022 agrees well with the value of 0.002 constrained by fluid/melt partitioning in an experimental analogue to volcanic degassing based on hydrous albite melts^62^.

### Numerical simulations

The model used to investigate the potential impact of the Minoan eruption on stratospheric chemical composition is a global two-dimensional (zonally averaged) chemistry-transport model that contains a detailed treatment of stratospheric aerosol microphysics. Its basic formulation is described elsewhere^71^,^72^. It extends from pole to pole and from the ground to 60 km with a horizontal resolution of 9.5 degrees (~1000 km) and a vertical resolution of half a pressure scale height (about 3.5 km). The model contains detailed representations of atmospheric chemistry (including wet/dry deposition) and transport. The sulphuric acid aerosol microphysics and the photochemical scheme for Ox, NOx, HOx, ClOx, BrOx, CHOx, and SOx are based on ref. 73. Gas phase reaction rates, photolysis cross-sections and heterogeneous reaction probabilities on sulfuric acid aerosol particles (including volcanic aerosols) follow the Jet Propulsion Laboratory recommendations^74^. The mean atmospheric circulation is calculated from forcing terms that include solar heating by O_2_ and O_3_ and longwave heating in the stratosphere by CO_2_, H_2_O, O_3_, CH_4_ and N_2_O. Thus the model includes important feedback processes between ozone, temperature, and transport in the stratosphere. Tropospheric heating rates and surface temperatures are specified.

## Additional Information

**How to cite this article**: Cadoux, A. *et al.* Stratospheric Ozone destruction by the Bronze-Age Minoan eruption (Santorini Volcano, Greece). *Sci. Rep.* **5**, 12243; doi: 10.1038/srep12243 (2015).

## Supplementary Material

Supplementary Information

## Figures and Tables

**Table 1:** Main characteristics of Santorini’s large silicic eruptions, volatile contents of glasses, and fractions released.

**Eruption**		**Minoan**	**Cape Riva**	**Lower Pumice 2**	**Lower Pumice 1**
Age	ky	3.6	22	172	184
Magma composition		rhyodacite	dacite	rhyodacite	rhyodacite
Magma density^*^	kg/m^3^	2308	2344	2318	2309
DRE volume^†^	km^3^	39	≥10 ?	>5.5 ?	5.5 ?
Mass of magma	kg	9 × 10^13^			
Plinian column height	km	36 ± 5	?	?	?
[S] matrix glass	ppm	100‡	63	47	60
[S] glass inclusions	“	104	99	50	101
[S] released by melt	“	4	35	3	41
[S] released by fluid	“	7900	6200	2260	7800
[F] matrix glass	“	***532***	558	514	698
[F] glass inclusions	“	798	812	796	872
[F] released by melt	“	266	255	282	174
[F] released by fluid	“	239	244	239	262
[Cl] matrix glass	“	2920‡	2725	2751	2663
[Cl] glass inclusions	“	3512	2781	2816	2843
[Cl] released by melt	“	592	56	65	180
[Cl] released by fluid	wt%	14	12	10	10
[Br] matrix glass^§^	ppm	10.70	9.98	10.08	9.75
[Br] glass inclusions^§^	“	12.86	10.19	10.32	10.41
[Br] released by melt	“	2.17	0.21	0.24	0.66
[Br] released by fluid	“	225	178	181	182
[I] matrix glass||	“	0.119	0.111	0.112	0.108
[I] glass inclusions||	“	0.143	0.113	0.115	0.116
[I] released by melt	“	0.024	0.002	0.003	0.007
[I] released by fluid	“	15	12	12	12

^*^Magma density calculated after the method of ref. 75

^†^DRE = Dense Rock Equivalent.

[i] denotes the concentration of volatile element i

Except for the values marked with a double dagger ‡ (ref. 21), volatile compositions are from ref. 16, values in Italic-bold are new measurements performed by EMP (Cameca SX 100, Clermont-Ferrand; same analytical procedure as that reported in ref. 16)

^§^Bromine contents estimated assuming a Cl/Br ratio of 273 (ref. 62)

|| Iodine contents estimated assuming a Br/I ratio of 90 (ref. 63)

Fraction of volatile released by the melt phase = [i] glass inclusion—[i] matrix glass

Fraction of volatile released by the fluid phase: see Methods. Volcanological and petrographical characteristics of the eruptions are further described in Supplementary Note.

**Table 2:** Apatite compositions, volatile partitioning and concentration of chlorine in the fluid.

**Eruption**	Minoan*	**SD**	**Cape Riva**	**SD**	**Lower Pumice 2**	**SD**	**Lower Pumice 1**	**SD**
**Sample #**			**S09–62**		**S09-23**		**S09–17**	
**n**	**22**		**10(15)**		**8(9)**		**8(9)**	
Composition (wt%)								
SiO_2_	0.23	0.15	0.30	0.25	0.26	0.04	0.23	0.05
CaO	54.18	0.84	54.60	0.36	54.66	0.29	54.71	0.35
P_2_O_5_	41.65	0.44	42.64	0.39	42.90	0.32	42.69	0.22
F	2.38	0.30	2.47	0.16	2.45	0.18	2.45	0.09
Cl	1.04	0.10	0.91	0.08	0.76	0.04	0.75	0.03
SO_3_	0.03	0.03	0.01	0.01	n.d.		0.01	0.01
Total	99.51		100.92		101.03		100.83	
Mole fractions of volatile components								
X_F_	0.63		0.66		0.65		0.65	
X_Cl_	0.15		0.13		0.11		0.11	
X_OH_	0.22		0.21		0.24		0.24	
Partition coefficients								
D_F_^ap/melt^	29.82		30.41		30.78		28.15	
D_Cl_^ap/melt^	2.96		3.26		2.70		2.64	
D_S_^ap/melt^	1.21		0.57				0.40	
Sructural formula								
Ca	9.59		9.49		9.48		9.52	
P	5.83		5.86		5.88		5.87	
Si	0.04		0.05		0.04		0.04	
Cl	0.29		0.25		0.21		0.21	
F	1.24		1.27		1.25		1.26	
OH	−0.53		−0.52		−0.46		−0.47	
Total	16.46		16.40		16.41		16.43	
Wt% Cl in fluid†	13.89		12.11		10.15		10.03	

n = number of crystal analysed (total number of analyses), SD = standard deviation.

n.d. = not detected.

Mole fractions of volatile components in hydroxyl site computed using the formulae of ref. 77.

X_F _, X_Cl_, X_OH_ = mole fraction of fluorapatite, chlorapatite and hydroxylapatite, respectively, in apatite.

D_i_^ap/melt^ = [i] in apatite divided by [i] in glass inclusions (i.e., pre-eruptive melt).

^*^Minoan apatite composition from ref. 76.

^†^Computed from the relationship X_Cl_ = 0.011 *(Wt% Cl in fluid[s]), established in ref. 27.

**Table 3:** Degassing budgets of 39 km^3^ of Minoan magma and measures of the atmospheric impact.

		**Minoan eruption**					**Man-made compounds**			
							**Late 1990s**		**Pre-industrial**	
		**S**	**F**	**Cl**	**Br**	**I**	**Cl**	**Br**	**Cl**	**Br**
Volatile yield estimates (Tg)	Min	0.34	22.7	50.6	0.1	0.002				
	Max	35.9	23.8	675	1.5	0.069	1			
Global atmospheric mixing ratios of Cl (in ppbv) and Br (in pptv)	Min			8	7.7					
	Max			106	103		3.8	20	0.55	5
EESC (ppt)	Min			5600						
	Max			74689						

Tg = Teragramme = 10^12^ g = 10^9^ kg = 10^6^ T = megatonne.

Cl and Br mixing ratios = number of moles of Cl and Br divided by the number of moles of air in the total

atmosphere (Na = 1.8*10^20^ moles of air) expressed as part per billion and part per trillion by volume of air

respectively.

EESC = Equivalent Effective Stratospheric Chlorine = [Cl] _added to stratosphere_ + 60 × [Br] _added to stratosphere_, calculated.

assuming that 10% reach the stratosphere (with 2.7*10^19^ moles of air) for sake of comparison with ref. 43.

Sources for anthropogenic compounds emissions and mixing ratios: refs 5, 45 & 46.
