# Supplementary material for: Stratospheric Ozone destruction by the Bronze-Age Minoan eruption (Santorini Volcano, Greece)
Source: Sci Rep. 2015 Jul 24;5:12243. doi: 10.1038/srep12243 (PMC4513290; doi:10.1038/srep12243)
Supplement: Supplementary Information [file srep12243-s1.pdf]

SUPPLEMENTARY INFORMATION

Stratospheric ozone destruction by the Bronze-Age Minoan eruption (Santorini Volcano, Greece)

Anita Cadoux*[1,2,3], Bruno Scaillet[1,2,3], Slimane Bekki[4], Clive Oppenheimer[5], Timothy H. Druitt[6]

[1] Université d'Orléans, ISTO, UMR 7327, 45071 Orléans, France.

[2] CNRS, ISTO, UMR 7327, 45071 Orléans, France.

[3] BRGM, ISTO, UMR 7327, BP 36009, 45060 Orléans, France.

[4] Sorbonne Universités, UPMC Université Paris 06 ; Université Versailles St-Quentin; CNRS/INSU, LATMOS-IPSL, France.

[5] University of Cambridge, Department of Geography, Downing Place, Cambridge CB2 3EN, United Kingdom.

[6] Laboratoire Magmas et Volcans, Université Blaise Pascal - CNRS - IRD, OPGC, 5 rue Kessler, 63038 Clermont-Ferrand, France.

*Corresponding author: acadoux@cnrs-orleans.fr

Supplementary Table 1| Calculation of the volatile yields of 39 km^3 of Minoan magma and atmospheric impacts

Mass of magma	[S] matrix	[S] GI	[S] fluid	Mass of fluid	Mass of S released by fluid	Mass of melt	Mass of S released by melt	Mass of S melt + fluid	Aerosol mass H_2SO_4 min	max
10^{12} g	ppm	ppm	ppm	g	g	g	g	g	g	g
89996	100	104	7900	4.5E+15	3.6E+13	8.5E+16	3.42E+11	3.59E+13	1.047E+12	1.099E+14
							Tg	Tg	Tg	Tg
							**0.34**	**35.9**	1.05	109.9

Mass of magma	[F] matrix	[F] GI	[F] fluid	Mass of fluid	Mass of F released by fluid	Mass of melt	Mass of F released by melt	Mass of F melt + fluid
10^{12} g	ppm	ppm	ppm	g	g	g	g	g
89996	532	798	239.40	4.5E+15	1.1E+12	4.5E+15	2.27E+13	2.38E+13
							Tg	Tg
							**22.7**	**23.8**

Mass of magma	[Cl] matrix	[Cl] GI	[Cl] fluid	Mass of fluid	Mass of Cl released by fluid	Mass of melt	Mass of Cl released by melt	Mass of Cl melt + fluid	Cl mixing ratio in the atmosphere		[Cl] added to the stratosphere		EESC		% rel. to pre-industrial EESC	
10^{12} g	ppm	ppm	ppm	g	g	g	g	g	**min**	**max**	**min**	**max**	**min**	**max**	**min**	**max**
89996	2920	3512	138854	4.5E+15	6.2E+14	4.5E+15	5.06E+13	6.75E+14	7.93E-09	1.06E-07	5.29E-09	7.06E-08				
							Tg	Tg	ppbv	ppbv	ppb	ppb	ppt	ppt		
							**50.6**	**675.0**	8	106	5.3	70.6	5600	74689	622	8299

Mass of magma	[Br] matrix	[Br] GI	[Br] fluid	Mass of fluid	Mass of Br released by fluid	Mass of melt	Mass of Br released by melt	Mass of Br melt + fluid	Br mixing ratio in the atmosphere		[Br] added to the stratosphere		% rel. to pre-industrial $[Br]_{stratosphere}$	
10^{12} g	ppm	ppm	ppm	g	g	g	g	g	**min**	**max**	**min**	**max**	**min**	**max**
89996	10.7	12.9	225.75	4.5E+15	1.0E+12	4.5E+15	1.88E+11	1.20E+12						
							Tg	Tg						
							**0.19**	**1.20**	pptv	pptv	ppt	ppt		
							**0.11***	**1.48***	7.7	103	5.2	68.8	87	1147

Mass of magma	[I] matrix	[I] GI	[I] fluid	Mass of fluid	Mass of I released by fluid	Mass of melt	Mass of I released by melt	Mass of I melt + fluid
10^{12} g	ppm	ppm	ppm	g	g	g	g	g
89996	0.12	0.14	14.87	4.5E+15	6.7E+10	4.5E+15	2.06E+09	6.90E+10
							Gg	Gg
							**2.1**	**69.0**

GI is for glass inclusions.

* Br yields directly obtained by multiplying the Cl yields with the Cl/Br ratio of 0.0022 (ref. 1, see Methods section)

Cl and Br mixing ratios in the total atmosphere (Na = $1.8*10^{20}$ moles of air) expressed as part per billion and part per trillion by volume, respectively
[Cl] and [Br] added to the stratosphere calculated assuming that 10% reach the stratosphere (with $2.7*10^{19}$ moles of air) for sake of comparison with ref. 2.

EESC = Equivalent Effective Stratospheric Chlorine = $[Cl]_{\text{added to stratosphere}} + 60 \times [Br]_{\text{added to stratosphere}}$ (refs. 3-5)

Pre-industrial EESC and [Br] are approximated on the basis of the WMO ozone assessment[5] as 900 ppt and 6 ppt, respectively, for pre-1980 values

Supplementary Note

The history of Santorini Volcano (South Aegean Arc, Greece) is marked by recurrent (every 20-30 ky) large-scale eruptions with Volcanic Explosivity Indices (VEI) $\geq$ 5. The Minoan (3.6 ka), Cape Riva (22 ka), Lower Pumice 2 (172 ka), and Lower Pumice 1 (184 ka) eruptions are amongst the largest Plinian eruptions of Santorini.

Eruptions magnitude and intensity of Santorini large Plinian eruptions

The size of the eruptions is difficult to gauge accurately, even for modern eruptions. The volume of erupted Minoan magma has been estimated to range between 28 km^3 (ref. 6) up to 39 km^3 (ref. 7) on the basis of terrestrial and deep-sea cores tephra deposits in the Eastern Mediterranean. More recently, submarine surveys of pyroclastic deposits around the volcano suggest that the emitted magma volume could be as large as 60 km^3 (ref. 8). Nevertheless, though it is clear that such deposits are derived from Santorini activity, their relationships to any given eruption remains to be further constrained, notably by comparing the chemical compositions of the juvenile components of this marine sequence to those of the terrestrial deposits. By including inferred intra-caldera products, Johnston et al.[9] recently estimated a total magma volume of 78-86 km^3 for the Minoan eruption. The maximum Plinian column height has been estimated to be 36 ± 5 km (refs. 10, 11). Modellings of Johnston et al.[12] indicate that the plume height of the last co-ignimbrite phase (phase 4; 27-40 km a.s.l) would be similar to that of the Plinian phase (phase 1).

The erupted magma volume of the Cape Riva eruption is at least about 10 km^3 (ref. 13). Volumes of the two Lower Pumice eruptions are poorly constrained, but must exceed a few km^3 each[14]. It is possible that the Lower Pumice 2 volume is at least as large as the Minoan[15].

Previous petrologic estimates of Santorini volatile budgets

Sulfur and chlorine emissions have been previously estimated for the Minoan and the Lower Pumice 2 eruptions using the conventional petrological method. The volatile budgets for the Cape Riva and Lower Pumice 1 eruptions have not been previously assessed.

Minoan rhyodacite

Using the conventional petrological method, Sigurdsson et al.[7] estimated that the maximum fraction of sulfur released during the Minoan eruption was about 65 ppm of the total erupted mass (8.4×10^{13} kg, 39 km^3 of dense rock equivalent of magma). This resulted in a sulfur yield to the atmosphere of 5.5×10^9 kg (i.e., 5.5 Tg). They considered that the chlorine yield was negligible because the chlorine content of melt inclusions and matrix glasses were similar (2578 ± 104 and 2745 ± 6 ppm, respectively). The more recent study Michaud et al.[16] reports:

(1) slightly lower chlorine contents in the matrix glasses than in the glass inclusions (2900 ± 250 and 3200 ± 150 ppm, respectively). Scaled to a magma mass of $6\text{-}8 \times 10^{13}$ kg, their estimate gives a chlorine release of $1.8\text{-}2.4 \times 10^{10}$ kg (i.e., 18-24 Tg).

(2) comparable sulfur contents in the glass inclusions and matrix glasses (90 ± 30 and 110 ± 10, respectively). This suggests negligible emission of sulfur during the eruption (ignoring thus the likely occurrence of a volatile phase in the reservoir). Their estimate of the sulfur yield to the atmosphere ($1.8\text{-}2.7 \times 10^{11}$ kg) is not directly comparable to that of ref. 7 because it includes the sulfur released during continuous passive degassing preceding the eruption. Thus, they considered the whole of the magmatic series, including basaltic magma, and assumed a much larger mass of magma (assuming 70% of crystallised parent magmas).

Lower Pumice 2 rhyodacite

Gertisser et al.[17] reported sulfur contents of 140 ± 50 ppm and 80 ± 20 ppm in the Lower Pumice 2 glass inclusions and matrix glasses, respectively. This gives a fraction of sulfur

released by the melt of about 60 ppm of the total mass of magma, which is comparable to the sulfur fraction estimated in ref. 23 for the Minoan eruption. Gertisser et al.[17] reported average chlorine contents of the glass inclusions (2490 ± 530 ppm), again comparable to that of the corresponding matrix glasses (2630 ± 210 ppm). According to these authors, the apparent lack of chlorine degassing from melt is consistent with their previous interpretations that the magmas involved in the LP2 eruption were water-undersaturated and any gas phase, if present, was CO_2-dominated, as chlorine would have partitioned strongly into a water-rich gas phase. They concluded that the chlorine emission to the atmosphere during the LP2 eruption was insignificant, even though some chlorine degassing could be inferred from the highest chlorine concentration of 3390 ppm they detected in the LP2 glass inclusions. Such an analysis ignores, however, the possible occurrence of brines in the magma, which would tend to buffer Cl content of the silicate melt at high values.

Supplementary Methods

Errors on S, Cl and Br yield estimates used in the numerical model

The total uncertainty associated with sulfur and chlorine emissions in the atmosphere mainly depends on errors in (1) the volume of magma emitted (i.e., the mass of magma), (2) measurements of S and Cl abundances in the volcanic glasses (i.e., in the pre-eruptive and post-eruptive melts), and (3) in the case of the presence of a pre-eruptive fluid phase (maximum estimate): the amount of fluid phase and the S and Cl concentrations in the fluid phase.

Error propagation (summing in quadrature) gives a relative error of 28 % for the estimates of S and Cl released by melt (i.e., 0.34 ± 0.10 and 50.6 ± 14.2 Tg, respectively), which is mainly due to the uncertainty on the 39 km^3 erupted magma volume (28 %, ref. 7). The relative error on our estimates of S and Cl released by melt + fluid is larger (up to 65% and 58%,

respectively) because of the large uncertainty (~50%) on the fraction of fluid in the pre-eruptive magma in addition to that of the erupted magma volume (and in the case of sulfur, to that of its estimated content in the fluid: ~30%; Methods).

When propagating the errors on Br estimates obtained using the average Br/Cl molar ratio of arc volcanoes gases[1], the relative error on the Cl yield estimate is added to the error on the value of Br/Cl (0.0022 ± 0.0009 within 95% confidence interval). Thus the relative errors on Br estimates exceed those of the Cl estimates by about 40%.

Estimation of the potential contribution of seawater-derived chlorine

Boiling of seawater during the phreatomagmatic phases (2 and 3) of the Minoan eruption may have released additional chlorine to the atmosphere. We assume that the boiled seawater vaporised all its Cl content. On the basis of Thermoremanent Magnetization (TRM) data[18] and heat conservation calculation, it is possible to estimate the volume of seawater involved in phases 2 and 3. Phase 4 was essentially dry and TRM data show that flows were emplaced at 300°C on average. The phase 2 and 3 flows were emplaced at temperatures up to 300°C (150°C on average). So it is reasonable to assume that the difference in emplacement T (150 °C) was due to loss of heat from the magma to seawater.

We took the following figures:

- Density of magma: 2300 kg/m^3
- Density of water: 1000 kg/m^3
- Specific heat of magma: 1000 J/kgK
- Specific heat of water: 4184 J/kgK
- Latent heat of vaporization of water: 2.26 x 10^6 J/kg

Phase 2 and Phase 3 are about 1 km^3 DRE (considering a total erupted volume of 39 km^3 DRE). Heat balance calculation gives a maximum volume of seawater involved in phases 2 and 3 of 0.12 km^3.

Given that the salinity of seawater is 3.5 wt%, this is equivalent to 4.36 x 10^9 kg of NaCl (assuming that all the sea salt is present as NaCl). Thus, the maximum seawater-derived Cl yield during the Minoan eruption would be 2.65 Tg, which is negligible in comparison with the 51-675 Tg of Cl released by the magma.

Supplementary Discussion

New insights into potential recordings of the Minoan eruption

It has been commonly believed that the Minoan eruption, which ejected ash and gases up to 36 km in the atmosphere, had an important environmental impact at a global scale and thus should have been recorded in tree rings and in Arctic glaciochemical records. Despite an uncertainty in the sulfur yield[19], some ice-core layers with high acidity peaks have been attributed to the Minoan eruption (e.g., 1636 BC peak in GRIP, Dye3 1645 BC signal, GISP2 1623 ± 36 BC signal; refs. 20-22). Nevertheless, several authors[7, 23-25] have questioned this correlation because of the discrepancy between the sulfur budget and ash chemistry recorded in the ice-core layers, on the one hand, and the petrological sulfur degassing estimates[7] and the Minoan ash composition, on the other hand. The sulfur degassing estimates of ref. 7 yielded a mass of H_2SO_4 aerosol in the 1645 BC layer (Dye3 IC) 6 to 12 times higher than the total H_2SO_4 aerosol yield of 1.7 x 10^{10} kg for the Minoan eruption.

However, this latter estimate was derived from the conventional petrological method (i.e., glass inclusion minus matrix glass sulfur contents), which represents a doubly minimum estimate as it is based on the assumption that (i) glass inclusions represent the pre-eruptive undegassed melt, and (ii) that the melt is the unique source of volatile degassing. Yet, our recent study[26] suggests that the pre-eruptive Minoan magma, as well as the magmas of the three other Santorini largest eruptions, were most probably saturated with a fluid phase (vapour and/or brine). The presence of a fluid phase increases dramatically the volatile

budgets[27], notably the total H_2SO_4 aerosol yield of the Minoan eruption (from 1 to 110 Tg; Supplementary Table 1).

The precise ^{14}C age of 1613 ± 13 BC (ref. 28) brings new perspectives and supports about the recording and the environmental impact of the eruption. It is in good agreement with dated tree ring anomalies throughout the NH and ice-core acidity layers in Greenland, which both have recorded volcanic events in the period 1617-1628 BC. Salzer & Hugues[29] identified tree rings (in Finland and Siberia) showing characteristics consistent with volcanic effects in the years 1619/1618/1617 BC. This matches well with an acidity layer in the GRIP ice-core, dated at 1618 BC (ref. 30). It is notable that some environmental events in China, dated approximately to 1618 BC, and suggestive of a volcanic winter, are reported in the Bamboo Annals (chronicle of ancient China): "yellow fog, a dim sun, then three suns, frost in July, famine, and the withering of all five cereals". Other tree rings precisely date the climatic effects of an eruption in 1626-1628 BC, the evidence including ring-width minima and frost-damaged rings in bristlecone pines of the western USA; bog oaks of Ireland, and other trees in Finland and Siberia (ref. 29 and references therein). This tree-ring date is consistent with two volcanic signals in the GISP2 and Dye3 ice-cores at 1623 (ref. 21) and 1622 BC (ref. 30), respectively. In the light of this study, future works on those ice-core layers could focus on HCl signals, as this could indicate if one of these glaciochemical anomalies may represent the Minoan eruption.

About potential climate impact of the Minoan eruption

The Bronze Age Minoan eruption of Santorini is one of the largest eruptions of the Holocene. In terms of magnitude (6.9-7.2, i.e., 30-60 km^3 of dense magma[19]), it is comparable to the 1815 Tambora eruption in Indonesia (30 km^3; ref. 31) whose global climatic and human impacts have been comprehensively documented[32]. However, the two events differ in their location: the 1815 Tambora eruption affected the global atmosphere due to its tropical

location, whilst our simulations (Figs. 1 & 2) show that the Minoan eruption primarily impacted the NH, as expected for a NH mid-latitude eruption. Yet, as for other large explosive eruptions, the expected climatic impact of the Minoan is also a decrease of globally- and annually-averaged temperature. The common method used to estimate this decrease for past eruptions is based on their sulfur output and the calculation of the sulfate aerosol radiative forcing (RF, in $W.m^{-2}$). The Minoan eruption's minimum sulfur yield (0.34 Tg; Table 3) is similar to that of the eruptions of Mt St Helens in 1980 and Mt Pelée in 1902 (0.34 and 1 Tg of S, respectively; ref. 27), neither of which had significant global effects[33]. However, our maximum sulfur yield estimate for the Minoan eruption (36 Tg) is comparable to that of the 1815 Tambora eruption (< 30 Tg; refs. 27 & 31) for which Zanchettin et al.[34] modelled a top of the atmosphere peak global mean sulfate aerosol RF of –4.5 $W.m^{-2}$ about a year after the eruption, resulting in a global near-surface air temperature decrease of up to 1.0°C. Notwithstanding the different latitudes of the two eruptions, Tambora's climatic effects give some guide to the potential magnitude of the Minoan eruption for the high SO_2 scenario. However, these cases neglect the potential climate forcing associated with pronounced stratospheric ozone losses following a large volcanic eruption. Ozone is a radiatively important gas as it absorbs both shortwave solar and longwave surface radiation. Recent estimates for RF associated with modern stratospheric ozone depletion range between –0.03 and –0.11 $W.m^{-2}$, with a mean of about –0.05 $W.m^{-2}$ (refs. 35- 39). This negative RF (i.e., cooling) has offset some of the warming due to increased greenhouse gas levels over the last few decades. Further studies using a suitable radiative transfer model[39] would be required to (i) constrain the relative magnitudes of surface cooling associated with sulfate aerosol and ozone loss, and (ii) to accurately determine the climatic impact of the Minoan eruption.

Supplementary references

1. Gerlach, T. M. Volcanic sources of tropospheric ozone-depleting trace gases. *G-cubed* **5**, Q09007, doi:10.1029/2004GC000747 (2004).

2. Kutterolf, S. *et al.* Combined bromine and chlorine release from large explosive volcanic eruptions: A threat to stratospheric ozone? *Geology* **41**, 707–710, doi:10.1130/g34044.1 (2013).

3. Danilin, M. *et al.* Bromine-chlorine coupling in the Antarctic ozone hole. *Geophys. Res. Lett.***23**, 153–156, doi:10.1029/95GL03783 (1996).

4. Sinnhuber, B.-M., Chipperfield, M. P. & Feng, W. The contribution of anthropogenic bromine emissions to past stratospheric ozone trends: A modelling study. *Atmos. Chem. Phys.* **9**, 2863–2871, doi:10.5194/acp-9-2863-2009 (2009).

5. Montzka, S. *et al.* Ozone-depleting substances (ODSs) and related chemicals. 1.1–1.86 (World Meteorological Organization, Geneva, 2011).

6. Pyle, D. M. *Thera and the Aegean world III: Vol. 2, Earth Sciences* [Hardy D., et al. (eds.)] [113-121] (The Thera Foundation, London, 1990).

7. Sigurdsson, H., Carey, S. & Devine, J. D. *Thera and the Aegean world III: Vol. 2, Earth Sciences* [Hardy D., et al. (eds.)] [100-112] (The Thera Foundation, London, 1990).

8. Sigurdsson, H. *et al.* Marine investigations of Greece's Santorini Volcanic Field. *Eos, Trans. Am. Geophys. Union* **87**, 337-342 (2006).

9. Johnston, E. N., Sparks, R. S. J., Phillips, J. C. & Carey, S. Revised Estimates for the Volume of the Late Bronze Age Minoan Eruption, Santorini, Greece. *J. Geol. Soc., London* **171**, 583-590, doi:10.1144/jgs2013-113 (2014).

10. Carey, S. N. & Sigurdsson, H. The intensity of Plinian eruptions. *Bull. Volc.* **51**, 28–40 (1989).

11. Sparks, R. S. J. & Wilson, C. J. N. *Thera and the Aegean world III: Vol. 2, Earth Sciences* [Hardy D., et al. (eds.)] [89–99] (The Thera Foundation, London, 1990).

12. Johnston, E. N., Phillips, J. C., Bonadonna, C. & Watson, I. M. Reconstructing the tephra dispersal pattern from the Bronze Age eruption of Santorini using an advection-diffusion model. *Bull. Volc.* **74**, 1485-1507 (2012).

13. Fabbro, G. N., Druitt, T. H. & Scaillet, S. Evolution of the crustal magma plumbing system during the build-up to the 22-ka caldera-forming eruption of Santorini (Greece). *Bull. Volc.* **75**, 1-22 (2013).

14. Druitt, T. H. et al. *Santorini Volcano*. (Geological Society, London, Memoirs, No. 19, 1999).

15. Keller, J., Gertisser, R., Reusser, E. & Dietrich, V. Pumice deposits of the Santorini Lower Pumice 2 eruption on Anafi island, Greece: Indications for a Plinian event of exceptional magnitude. *J. Volcanol. Geoth. Res.* **278-279**, 120-128 (2014).

16. Michaud, V., Clocchiatti, R. & Sbrana, S. The Minoan and post-Minoan eruptions, Santorini (Greece), in the light of melt inclusions: chlorine and sulphur behaviour. *J. Volcanol. Geoth. Res.* **99**, 195-214 (2000).

17. Gertisser, R., Preece, K. & Keller, J. The Plinian Lower Pumice 2 eruption, Santorini, Greece: Magma evolution and volatile behaviour. *J. Volcanol. Geoth. Res.* **186**, 387-406 (2009).

18. McClelland, E. & Thomas, R. *Thera and the Aegean world III: Vol. 2, Earth Sciences* [Hardy D., et al. (eds.)] [129-138] (The Thera Foundation, London, 1990).

19. Oppenheimer, C. in *4th Archaeological Conference of Central Germany.* (eds H. Meller, F. Bertemes, & H.R. Bork) 49-58 (2013).

20. Hammer, C. U., Clausen, H. B., Friedrich, W. L. & Tauber, H. The Minoan eruption of Santorini in Greece dated to 1645 BC? *Nature* **328**, 517-519 (1987).

21. Zielinski, G. A. *et al.* Record of volcanism since 7000 B.C. from the GISP2 Greenland ice core and implications for the volcano–climate system. *Science* **264**, 948–952 (1994).

22. Vinther, B. M. *et al.* A synchronized dating of three Greenland ice cores throughout the Holocene. *J. Geophys. Res.: Atmospheres* **111**, D13102 (2006).

23. Zielinski, G. A. & Germani, M. S. New ice-core evidence challenges the 1620s BC age for the Santorini (Minoan) eruption. *J. Archeo. Sci.* **25**, 279–289 (1998).

24. Keenan, D. J. Volcanic ash retrieved from the GRIP ice core is not from Thera. *G-cubed* **4**, 1-8, doi:10.1029/2003GC000608 (2003).

25. Pearce, N. J. G. *et al.* Identification of Aniakchak (Alaska) tephra in Greenland ice core challenges the 1645 BC data of Minoan eruption of Santorini. *G-cubed* **5**, Q03005, doi:10.1029/2003GC000672 (2004).

26. Cadoux, A., Scaillet, B., Druitt, T. H. & Deloule, E. Magma storage conditions of large Plinian eruptions of Santorini Volcano (Greece). *J. Petrol.* **55**, 1129-1171, doi:10.1093/petrology/egu021 (2014).

27. Scaillet, B., Luhr, J. F. & Carroll, M. C. *Volcanism and the Earth's Atmosphere, Geophysical Monograph* [Robock, A. & Oppenheimer, C. (eds.)] [11-40] (American Geophysical Union, 2003).

28. Friedrich, W. L. *et al.* Santorini Eruption Radiocarbon Dated to 1627–1600 BC. *Science* **312**, 548, doi:10.1126/science.1125087 (2006).

29. Salzer, M. W. & Hughes, M. K. Bristlecone pine tree rings and climatically-effective volcanic eruptions over the last 5000 years. *Quat. Res.* **67**, 57-68 (2007).

30. Clausen, H. B. *et al.* A comparison of the volcanic records over the past 4000 years from the Greenland Ice Core Project and Dye 3 Greenland ice cores. *J. Geophys. Res.: Oceans* **102**, 26707-26723 (1997).

31. Self, S. *et al.* Magma volume, volatile emissions, and stratospheric aerosols from the 1815 eruption of Tambora. *Geophys. Res. Lett.* **31**, L20608 (2004).

32 Oppenheimer, C. Climatic, environmental and human consequences of the largest known historic eruption: Tambora volcano (Indonesia) 1815. *Progress in Physical Geography* **27**, 230-259, doi:10.1191/0309133303pp379ra (2003).

33. Robock, A. The Mount St. Helens Volcanic Eruption of 18 May 1980: Minimal Climatic Effect. *Science* **212**, 1383-1384, doi:10.1126/science.212.4501.1383 (1981).

34. Zanchettin, D. *et al.* Background conditions influence the decadal climate response to strong volcanic eruptions. *J. Geophys. Res.: Atmospheres* **118**, 4090–4106, doi:10.1002/jgrd.50229 (2013).

35. Forster, P. M., et al. *Climate Change 2007: The Physical Science Basis. Contribution of Working Group I to the Fourth Assessment Report of the Intergovernmental Panel on Climate Change* [Solomon, S. *et al.* (eds)] (Cambridge University Press, 2007).

36. Scientific Assessment of Ozone Depletion: 2010. Report No. 52, 517 (World Meteorological Organization, Geneva, Switzerland, 2010).

37. Cionni, I., *et al.* Ozone database in support of CMIP5 simulations: Results and corresponding radiative forcing. *Atmos. Chem. Phys.* **11**, 11,267–11, 292 (2011).

38. Hassler, B., *et al.* Comparison of three vertically resolved ozone databases: Climatology, trends and radiative forcings. *Atmos. Chem. Phys.* **12**, 26,561–26,605 (2012).

39. Bekki, S. *et al.* Climate impact of stratospheric ozone recovery. *Geophys. Res. Lett.* **40**, 2796-2800 (2013).
